# Bioconversion of Lignocellulosic Materials with the Contribution of a Multifunctional GH78 Glycoside Hydrolase from *Xylaria polymorpha* to Release Aromatic Fragments and Carbohydrates

**DOI:** 10.4014/jmb.2106.06053

**Published:** 2021-08-20

**Authors:** Christiane Liers, René Ullrich, Harald Kellner, Do Huu Chi, Dang Thu Quynh, Nguyen Dinh Luyen, Le Mai Huong, Martin Hofrichter, Do Huu Nghi

**Affiliations:** 1 International Graduate School of Zittau (IHI Zittau), Dresden University of Technology, D-03583 Zittau, Germany; 2 Department of Cellular and Molecular Anatomy, Hamamatsu University School of Medicine, Hamamatsu, Shizuoka 431-3192, Japan; 3 Institute of Natural Products Chemistry, Vietnam Academy of Science and Technology, 18 Hoang Quoc Viet, Hanoi, Vietnam; 4 Graduate University of Science and Technology, Vietnam Academy of Science and Technology, 18 Hoang Quoc Viet, Hanoi, Vietnam

**Keywords:** Glycoside hydrolase, enzyme cocktail, ascomycetous fungus, *Xylaria polymorpha*, lignocelluloses

## Abstract

A bifunctional glycoside hydrolase GH78 from the ascomycete *Xylaria polymorpha* (*Xpo*GH78) possesses catalytic versatility towards both glycosides and esters, which may be advantageous for the efficient degradation of the plant cell-wall complex that contains both diverse sugar residues and esterified structures. The contribution of *Xpo*GH78 to the conversion of lignocellulosic materials without any chemical pretreatment to release the water-soluble aromatic fragments, carbohydrates, and methanol was studied. The disintegrating effect of enzymatic lignocellulose treatment can be significantly improved by using different kinds of hydrolases and phenoloxidases. The considerable changes in low (3 kDa), medium (30 kDa), and high (> 200 kDa) aromatic fragments were observed after the treatment with *Xpo*GH78 alone or with this potent cocktail. Synergistic conversion of rape straw also resulted in a release of 17.3 mg of total carbohydrates (*e.g.*, arabinose, galactose, glucose, mannose, xylose) per gram of substrate after incubating for 72 h. Moreover, the treatment of rape straw with *Xpo*GH78 led to a marginal methanol release of approximately 17 μg/g and improved to 270 μg/g by cooperation with the above accessory enzymes. In the case of beech wood conversion, the combined catalysis by *Xpo*GH78 and laccase caused an effect comparable with that of fungal strain *X. polymorpha* in woody cultures concerning the liberation of aromatic lignocellulose fragments.

## Introduction

Lignocellulose-containing biomass, the largest renewable reservoir of potentially fermentable carbohydrates produced by plant photosynthesis, has gained growing interest as a sustainable alternative to fossil carbon resources to produce 2^nd^-generation biofuels and other biobased chemicals. Thus, as raw material for biotechnological and industrial applications, this vast resource becomes more and more economically important, not least against the background of intensified biomass utilization in the sense of the biorefinery concept and the idea of sustainable development [1, 2]. Lignocellulose comes from plant synthesis of complex cell-wall polymers and provides the rigidity and mechanical stability to protect plants from microbial attack. The particular properties of lignocellulose are based on the structure of its major components - cellulose (backbone), hemicelluloses (xylan; covering material), and lignin (molecular glue) - which are strongly intermeshed and chemically bonded and therefore limit the process of hydrolysis [3]. In addition, pectin is also the major matrix component within the lignocellulose of cell walls, especially in non-woody fibers. However, it is usually not found in wood tissues because the secondary wall thickening replaces almost all pectin with lignin [4].

Since the process requires less energy in mild conditions, enzymatic hydrolysis is becoming a suitable pathway in biomass hydrolysis. Moreover, due to lignocellulosés rigidity and mechanical stability, critical to successful use of this biomass is the development of enzyme cocktails that will break the plant cell wall down into usable fractions [3, 5]. In addition to some well-described oxidative enzymes (*e.g.*, laccase, lignin peroxidase, manganese peroxidase) involved in lignocellulose degradation [6-8], several hydrolytic enzymes belonging to glycoside hydrolases and carbohydrate esterases are needed for a complete conversion [9, 10]. Glycoside hydrolases (GHs; or glycosidases, EC 3.2.1.x) catalyze the hydrolysis of a wide variety of glycosidic linkages between two or more carbohydrates or between a carbohydrate and a non-carbohydrate moiety. Although hundreds of GHs (*e.g.*, cellulases, β-xylanases, β-mannanases, β-galactosidases, α-L-rhamnosidase, etc.) from both fungal and bacterial sources have been identified and biochemically characterized, the search continues for new GHs with better properties regarding enhanced activity, selectivity, and pH/thermostability, as well as for multifunctional enzymes with industrial applications [5, 11].

In a previous study, we focused on the isolation as well as the catalytic and molecular characterization of a novel GH78 glycoside hydrolase (belonging to the GH78 family according to its sequence) from the wood rot ascomycete *X. polymorpha* (designated as *Xpo*GH78), which exhibited both α-L-rhamnosidase and feruloyl esterase activities [12]. Thus, *Xpo*GH78 is the first fungal enzyme of the GH78 family to exhibit alkyl-aryl esterase activity on numerous natural and synthetic esters and enable the soft rot fungus to hydrolyze the lignocellulosic complex partially. Such a catalytic versatility combined in one protein with activities towards both glycosides and esters may be advantageous for the efficient degradation of the plant cell-wall complex that contains both diverse sugar residues and esterified structures. Based on this research, it will be worthwhile to use this multifunctional hydrolytic enzyme alone and in combination with accessory hydrolases and oxidase ("enzyme cocktail") for the efficient conversion of lignocellulosic materials to release *e.g.*, water-soluble aromatic fragments, carbohydrates, and biomethanol without any chemical pretreatment.

## Materials and Methods

### Materials

Lignocellulosic sources such as birch wood, beech wood, wheat bran, wheat straw, rape straw, birchwood xylan, beech wood xylan, oat spelt xylan, and wheat arabinoxylan were provided by Sigma-Aldrich (Germany) and Megazyme (Ireland). Hydrolase preparation (cellulase and xylanase activities) from *Trichoderma reesei* was obtained from AB Enzymes (Germany). The purified laccase from *X. polymorpha* (128 U/mg) was provided by R. Ullrich (IHI Zittau).

### Fungus and Growth Conditions

The wood decay ascomycete *Xylaria polymorpha* was obtained from the Department of Environmental Biotechnology (IHI Zittau). The stock culture was maintained at 4°C on malt extract agar (MA) plates containing 20 g malt extract per liter after fungal mycelia fully covered the agar surface. The pre-culture was prepared by transferring an agar plug (∅ 1cm) from stock culture onto a new MA plate and then incubated at 23°C. The 3-week-old pre-cultures readily served for the subsequent experiments.

For enzyme production on solid-state culture, approximately 2 kg wheat straw was pre-soaked with distilled water overnight and filled in a 10-L autoclavable plastic bag (H+P Labortechnik, Germany). After sterilization (121°C for 30 min), inoculation was performed using two overgrown MA plates of *X. polymorpha* and incubated at 23°C for 6-8 weeks.

### Liquid Cultivation

Firstly, a minimal medium containing the essentials for fungal growth (0.5 g MgSO_4_, 1.5 g KH_2_PO_4_, 2.0 g/l yeast extracts) was prepared. Thereafter, 100-ml Erlenmeyer flasks containing 50 ml of the above minimal medium (pH 6.0) each were supplemented with 2% (w/v) of potential feruloyl esterase-stimulating substances, *i.e.*, different ester- and lignocellulosic sources: birch wood, beech wood, wheat bran, wheat straw, rape straw, birchwood xylan, beech wood xylan, oat spelt xylan, and wheat arabinoxylan as well as triesters: triacetin and olive oil. Afterward, 5 ml of the suspension was transferred into a liquid medium for incubation at 23°C on a rotary shaker (200 rpm). During incubation of 21 days, aliquots (1 ml) were taken from fungal liquid cultures after intervals of 3 days for the measurement of esterase activity.

### Enzyme Assays

Feruloyl esterase activity of *Xpo*GH78 was determined by hydrolytic demethylation of 1 mM methyl ferulate to the ferulic acid in 3-(*N*-morpholino)propane sulfonic acid (100 mM MOPS) buffer at pH 6.0. The reaction was initiated by the incubation of reaction mixtures at 37 ° for a suitable time depending on the enzyme sample (10-30 min) and then terminated by an equal volume of acetic acid/acetonitrile (11.3%; v/v) as a stop solution [13]. After centrifugation, the released ferulic acid was analyzed by HPLC as described below.

Laccase activity was measured by the oxidation of 2,2’-azino-*bis*(3-ethylbenzthiazonline-6-sulphonic acid)(ABTS) to the corresponding cation radical [ABTS]^+•^ as previously described [14].

### Isolation and Purification of *Xpo*GH78

The purification of *Xpo*GH78 was performed using an FPLC ÄKTA system (GE, Germany). The enzymatic extract from *X. polymorpha* culture was applied to different anion-exchange and size-exclusion chromatography steps as described previously [12].

### Hydrolysis of Beech Wood and Rape Straw by an Enzyme Cocktail Containing *Xpo*GH78

Enzymatic reactions were performed under conditions as the previous study [12], in which the temperature and pH optima of *Xpo*GH78 were found to be 37-45°C, pH 6-8 and incubation time for the conversion of the lignocellulosic materials to be for 48 to 72 h, respectively.

The wheat straw and beech wood materials were ground to a fine powder (< 200 μm in size; determined microscopically) by a planetary ball mill (Fritsch, Germany), suspended in distilled water (1%; w/v), and then used as stock solution. The different conditions were set based on the specific properties of materials (*i.e.*, wood meal or straw material) and the product analysis as follows:

The beechwood meal (0.5%; w/v) was incubated with enzymes from *X. polymorpha* (0.1 U *Xpo*GH78 and 1 U *Xpo*Lac) for 48 h at 37°C. The reaction was carried out in 100 mM MOPS buffer (pH 6.0), and for comparison purposes, a control with heat-inactivated enzyme (95°C for 30 min) was used.

Hydrolysis of finely milled rape straw was investigated using *Xpo*GH78 and further hydrolases and oxidoreductases, which were commercially available (AB Enzymes) or previously purified, to improve the efficiency of the conversion process. Rape straw material (3 g/reaction) was in vitro incubated with *Xpo*GH78 (0.4 U/mg) and selected enzymes in 100 mM sodium citrate buffer at pH 6.0. The commercially available preparation of AB Enzymes is a mixture of different recombinant hydrolases produced by *T. reesei* (carboxymethyl cellulase 1.6 U/mg and glucuronoxylanase 1 U/mg).

The reactions, a total of 30 ml of each containing the above components in 50-ml tubes, were incubated at 37°C for 72 h. To assess the synergistic and single effects of each enzyme, the enzymatic conversion was performed with each enzyme alone and in combinations on the one hand with all hydrolases (*Xpo*GH78 and AB Enzymes preparation) and on the other hand, with all tested enzymes (*Xpo*GH78, AB Enzymes preparation, and laccase). Controls containing heat-denatured enzymes (95°C for 15 min) were used.

### Gas Chromatography-Tandem Mass Spectrometry (GC-MS)

The GC-MS method was applied to estimate the enzymatic demethylation of rape straw by *Xpo*GH78 alone or by enzyme cocktails. After incubation of corresponding substrates under the conditions described above, the release of methanol by enzymatic hydrolysis was analyzed using a GC-MS system (Hp 6890 series, Agilent, Germany) fitted with a Zebron column (ZB-WAXplus, 250 μm × 30 m, 0.25 μm film thickness, Phenomenex®, Germany). The data were acquired according to GC retention time and electron impact MS in the selected ion monitoring (SIM, 31 m/z) mode at 70 eV [15].

### High-Performance Liquid Chromatography (HPLC)

Reaction aliquots were briefly centrifuged (12,000 ×*g*) and then transferred into 1.5-ml HPLC vials. For analysis of ferulic acid, substances were routinely eluted on a reversed-phase C_18_-column (Synergi Fusion-RP 80A, 4.6 mm × 125 mm, USA) using an Agilent HPLC system (1200 series) equipped with a diode-array detector (DAD) at wavelengths of 323 nm. For monosaccharide analysis, a Rezex HPLC ion-exclusion column [RPM-Monosaccharide Pb^+2^ (8%), 7.8 mm × 300 mm, Phenomenex], and a refractive index detector (RID) were used.

### High-Performance Size-Exclusion Chromatography (HPSEC)

HPSEC was used to determine the molecular mass distribution of lignocellulose fragments formed after enzymatic action on milled beech wood. It was performed on an HPLC system (HP 1200 Liquid Chromatography, Agilent) fitted with a HEMA-Bio linear column (8 mm × 300 mm, Polymer Standard Service, Germany). The elution at pH 10.0 by the mobile phase consisting of 20% acetonitrile and 80% of an aqueous solution (0.34% NaCl and 0.2% K_2_HPO_4_) was monitored at 280 nm by a DAD detector. Sodium polystyrene sulfonates (1.3-168 kDa, Polymer Standard Service) and biphenyl dicarboxylic acid (0.246 kDa) served as molecular mass standards [16, 17].

## Results

**Fig. 1:**
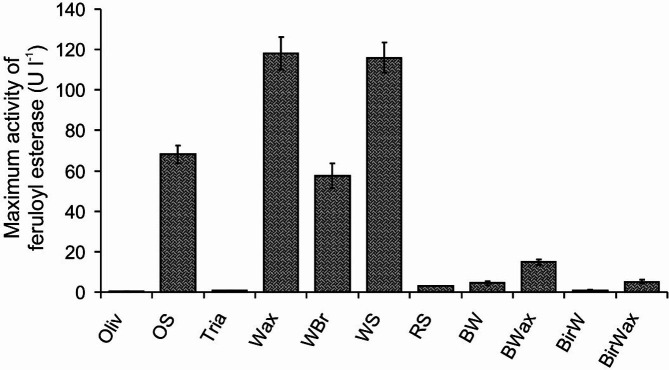
Overview of the maximum activities of multifunctional GH78 glycoside hydrolase (*Xpo*GH78) from *X. polymorpha* in liquid cultures during 21-day incubation with potential inducing agents: olive oil (*Oliv*), oat spelt xylan (*OSXyl*), triacetin (*Tria*), wheat arabinoxylan (*Wax*), wheat bran (*WBr*), wheat straw (*WS*), rape straw (*RS*), beech wood (*BW*), beech wood xylan (*BXyl*), birch wood (*BirW*), and birchwood xylan (*BirXyl*). Aliquots were collected at time intervals of 3 days for enzyme activity measurement (*error bars*).

Regarding the feruloyl esterase activity of the multifunctional GH78 glycoside hydrolase *Xpo*GH78, the ascomycetous strain *X. polymorpha* was grown in liquid cultures supplemented with different lignocellulosic substances consisting of ester bonds and serving as the sole carbon source. As shown in Fig. 1, wheat straw, wheat arabinoxylan, wheat bran, and oat spelt xylan remarkably stimulated the hydrolytic production in comparison to the control, which did not contain any inducer and none of the esterase activities. At the applied concentrations of wheat straw and wheat arabinoxylan (2%), the highest levels (~ 120 U/l) of enzyme activities were obtained. Both substances seem to be the most suitable natural inducers for improved enzyme production. At the same concentration, oat spelt xylan and wheat bran also had an enhancing effect that turned out to be approximately half of the maximal attained activities of wheat straw substrates (~ 60 U/l). Other tested carbon sources such as triacetin and olive oil did not effectively stimulate the respective enzyme production.

For larger-scale enzyme production used for further enzymatic conversion of biomass, the straw-like substrate was chosen, and a multifunctional GH78 glycoside hydrolase (*Xpo*GH78; a molecular mass of 98 kDa and a weak acidic *p*I value of 3.7) was purified from a culture of *X. polymorpha* regarding its feruloyl esterase activity as described previously by Nghi *et al*. [12].

### Enzymatic Conversion of Beech Wood to Release an Aromatic Fragment

**Fig. 2:**
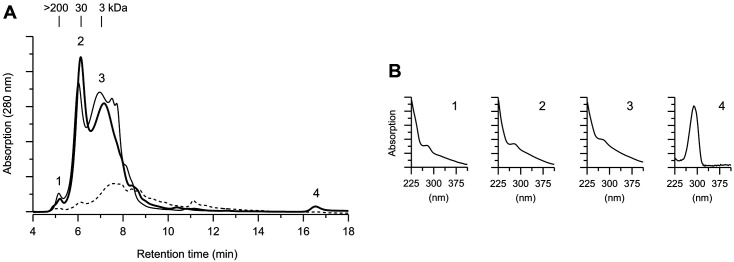
(**A**) HPSEC-elution profiles of the molecular weight distribution of water-soluble aromatic fragments released from beech wood after 48 h of in vitro incubation with a combination of *X. polymorpha* hydrolase (*Xpo*GH78; 0.1 U) and laccase (*Xpo*Lac; 1 U) (*bold line*) and from a 42-day aged beech wood culture of *X. polymorpha* (*solid line*). Control consisted of milled beech wood incubated without enzyme (*dashed line*). (**B**) UV/Vis spectra of corresponding lignocellulose fragments.

Purified *Xpo*GH78 and the *X. polymorpha* laccase (*Xpo*Lac) were used to convert milled beech wood material, which contains about 45.8 wt% cellulose 23.2 wt% hemicellulose, and 24.3 wt% lignin [18]. After 48 h of incubation, the release of medium-sized water-soluble aromatic fragments (3 and 30 kDa) from the wood meal was detected by HPSEC, as shown in Fig. 2. Furthermore, a high-molecular-mass fraction (molecular size > 200 kDa) was also found and could be assumed as a polymerization product formed by laccase-catalyzed oxidation of phenolics. The UV/Vis spectra of the released aromatic fragments indicated a lignin-like character with typical absorption maxima between 250 and 280 nm compared to water-soluble model lignin, *i.e.*, alkaline lignin and a lignin sulfonic acid. Interestingly, the elution profile of the in vitro conversion with both purified enzymes of *X. polymorpha* was similar and comparable to that of the 42-day aged in vivo culture of the whole fungus. Especially, the main fragments with a molecular size of 3, 30, and 200 kDa appeared in both chromatograms, probably indicating the enzyme influence during the fungal attack of the woody material. Not least, both enzymes could be detected in the aqueous extract of fungal cultures.

### Release of Water-Soluble Aromatic Fragments from Rape Straw

To demonstrate an effective conversion of lignocelluloses by the pure *Xpo*GH78 with and without accessory enzymes (hydrolases and oxidases), rape straw (the total contents of cellulose, hemicellulose, and lignin are about 49.5 wt%, 12.7 wt%, and 17.7 wt%, respectively; [19]) was chosen as the target substance. This substrate (3 g/reaction) was incubated at 23°C for 72 h with *Xpo*GH78 solely or in different combinations with other enzymes.

**Fig. 3:**
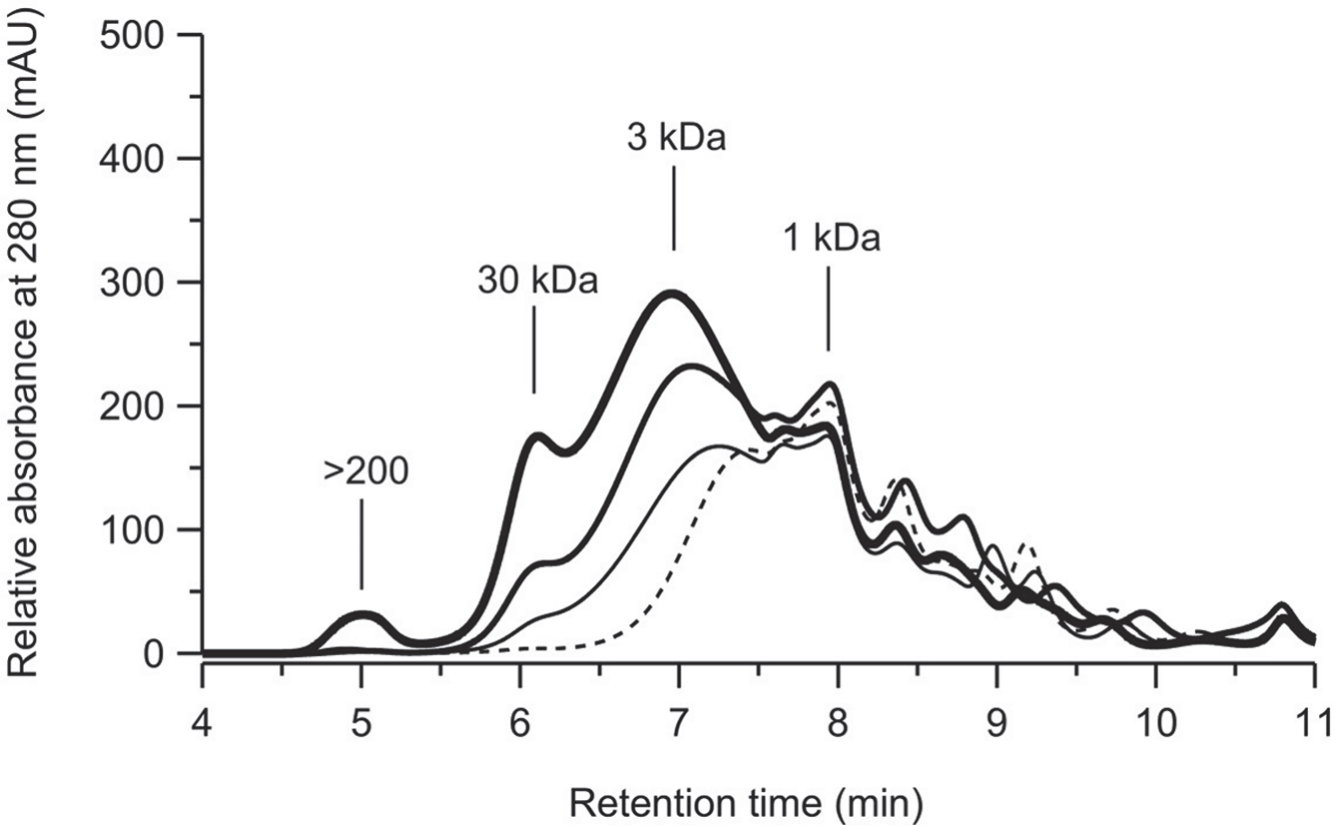
HPSEC-elution profile of water-soluble aromatic fragments (absorbance at 280 nm) released from rape straw after treatment with different enzymes: laccase (*Xpo*Lac; *thin line*), *X. polymorpha* hydrolase (*Xpo*GH78; *solid line*), and enzyme cocktails of *Xpo*GH78, *Xpo*Lac, and commercial enzyme preparation from AB Enzymes containing cellulose/xylanase activity (Cell/Xyl; *bold line*). Control (*dashed line*).

In Fig. 3, the changes in molecular weight distribution of water-soluble aromatic fragments caused by the different enzymatic reaction systems are shown. As a result, purified *X. polymorpha* laccase (*Xpo*Lac; spec. activity of 22 U/mg) alone caused a marginal change in the fragment pattern of water-soluble aromatics. In contrast, pure *Xpo*GH78 increased the release of fragments and changed the fragment pattern of the aromatics. Especially, the number of water-soluble fragments with a low and medium size (3 and 30 kDa) increased after the reaction with this new hydrolase. Interestingly, a slight increase at 1 kDa was observed, indicating a depolymerizing effect caused by the *Xpo*GH78. Compared to the modification of lignocellulose fragments caused by *Xpo*Lac and *Xpo*GH78, the highest efficiency was achieved with the reaction system containing additional cellulase and xylanase. The considerable changes in low (3 kDa), medium (30 kDa), and high (> 200 kDa) molecular fragments were observed after the treatment with this potent cocktail.

### Release of Carbohydrates from Rape Straw

A similar synergistic effect was observed to release sugars from rape straw meal caused by enzymatic treatment with single hydrolases or oxidases and in a combination of these enzymes. Table 1 shows the enzymatically released amounts of the five most abundant sugars in the cellulose and hemicellulose moieties of straw material after correction with the corresponding controls. Increasing amounts (4.7; 5.5; and 5.9 mg/g) could be measured to release glucose after treatment with the AB Enzymes preparation, the latter supplemented with *Xpo*GH78, and finally by using all enzymes together. Such a synergistic effect was also observed for galactose (0.5; 1.5; and 5.9 mg/g).

**Fig. 4:**
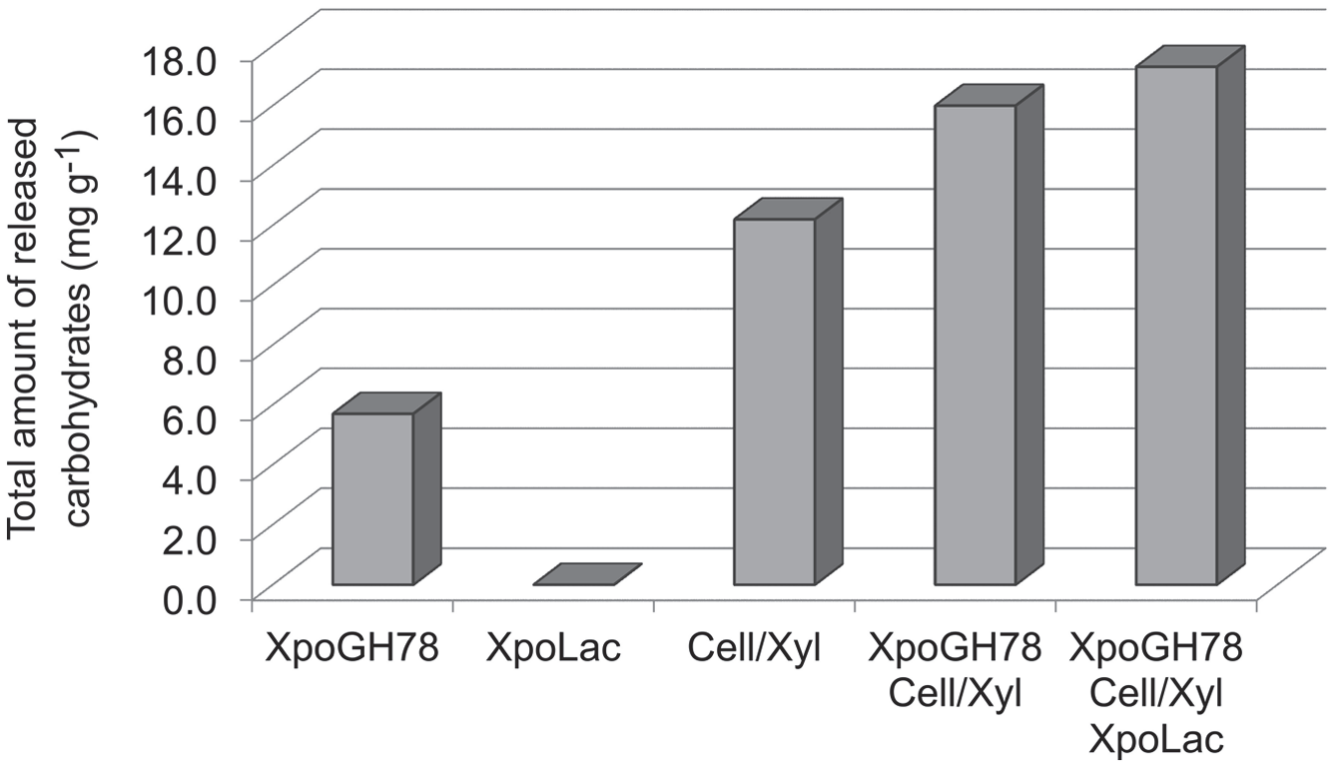
Sum of the released carbohydrates from milled rape straw after treatment with different enzymes (*Xpo*GH78, *Xpo*Lac, and Cell/Xyl) as single applications, and in two-enzyme combinations [(i) *Xpo*GH78 and Cell/Xyl; (ii) *Xpo*GH78, Cell/Xyl, and *Xpo*Lac]. The values are corrected by the sugar amounts in the corresponding controls containing heat-denatured enzymes.

The comparison of the sum of all five detected carbohydrates released by each enzymatic reaction system is shown in Fig. 4. The effect caused by the multifunctional *Xpo*GH78 (total amount of released carbohydrates: ~ 6 mg/g) is half of that observed for the cellulase/xylanase cocktail (Cell/Xyl; 12 mg/g). It is no surprise that the oxidase *Xpo*Lac alone did not affect sugar liberation from rape straw meal. Notably, the overall carbohydrate release increased up to 16 mg/g using a combination of *Xpo*GH78 and the crude cellulase/xylanase preparation. The addition of *Xpo*Lac to this enzyme cocktail containing only hydrolases further increased the effect up to 17.3 mg/g.

### Release of Methanol from Rape Straw

**Fig. 5:**
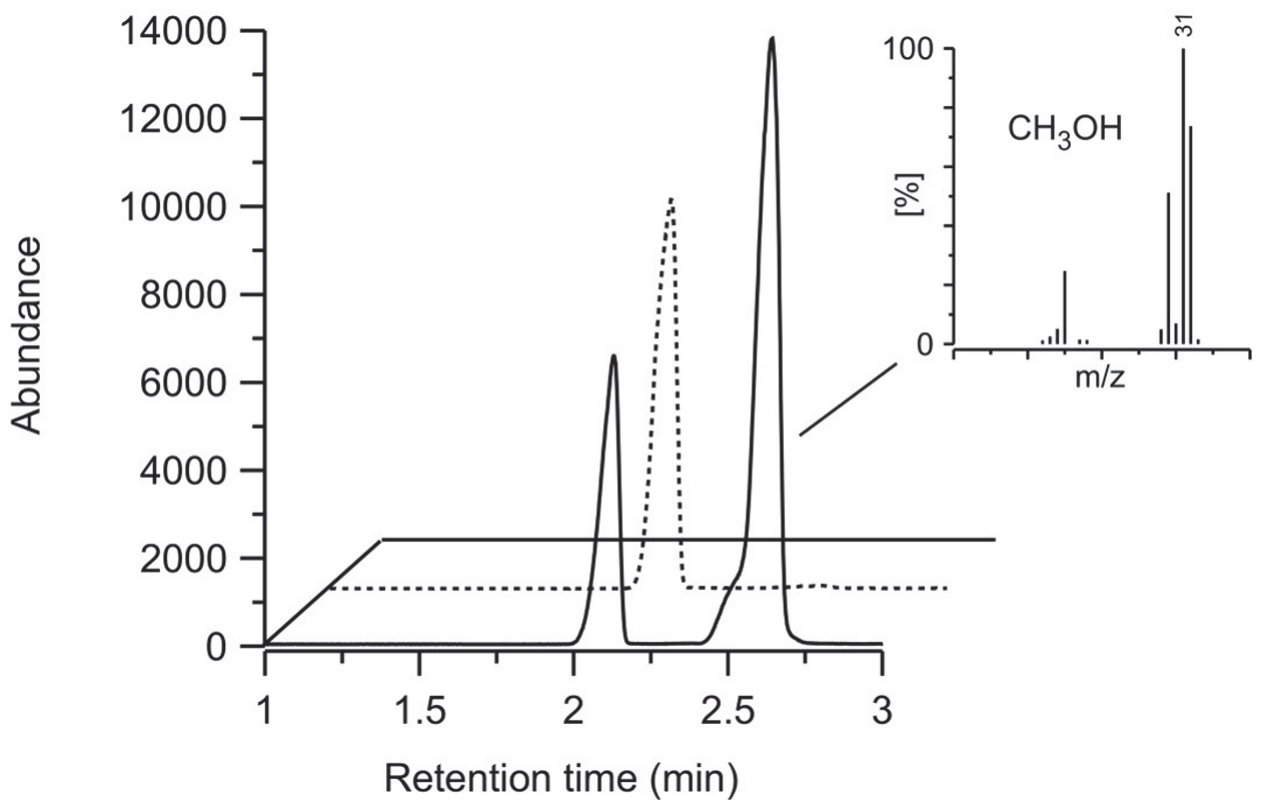
GC-MS elution profile of the enzymatic demethylation of rape straw (*solid line*) after 48 h of incubation. Control without enzyme (*dashed line*). Insert shows the mass spectrum of the reaction product methanol.

**Fig. 6:**
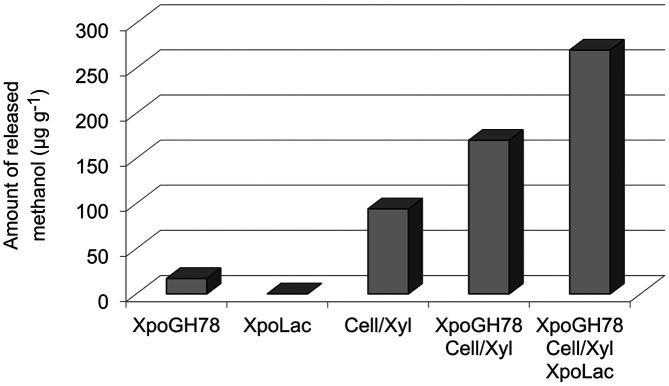
Release of methanol from milled rape straw after demethylation by different enzymes (*Xpo*GH78, *Xpo*Lac, and Cell/Xyl) as single applications, and in two combinations of enzyme systems [(i) *Xpo*GH78 & Cell/Xyl; (ii) *Xpo*GH78, Cell/Xyl, and *Xpo*Lac)].

Demethylation activity of *Xpo*GH78 on the methyl-groups occurring in the pectin moieties of rape straw was demonstrated by GC-MS detection of the reaction product methanol (Fig. 5). For that reason, the improvement of methanol release by the synergistic action of hydrolases and oxidases was investigated. The incubation of rape straw with *Xpo*GH78 for 72 h at 23°C led to a marginal methanol release of approx. 17 μg/g and with crude cellulose/xylanase to 94 μg/g. This effect could be considerably improved by combining both preparations together, which led to a methanol release of approximately 170 μg/g. The addition of *Xpo*Lac to this hydrolase cocktail finally increased the methanol release up to 270 μg/g (Fig. 6).

## Discussion

The ability of purified GH78 glycoside hydrolase (*Xpo*GH78) to hydrolyze lignocellulosic materials was tested for different materials such as milled beech wood and rape straw as well as for beech wood with and without the addition of accessory enzymes. As main metabolites, water-soluble aromatic fragments, carbohydrates, and methanol could be detected, which are all biotechnological interests. *Xpo*GH78 can act on beech wood meal in combination with the extracellular laccase of the same fungus. The elution profiles of the released aromatic, water-soluble fragments on the one hand caused by the enzymatic attack in vitro, and the aromatic fragments, on the other hand, extracted from a 42-day aged in vivo culture of *X. polymorpha* were found to be quite similar (3, 30, and 200 kDa-sized fragments in both cases). This could be evidence for the synergistic effect of hydrolase and laccase, which are both active during the fungus growth on beech wood chips [12, 14].

The fragment pattern of soluble aromatics released after enzymatic treatment of rape straw meal with *Xpo*GH78 combined with further hydrolytic and oxidative enzymes is comparable with that of the in vivo conversion of beech wood. Accordingly, the characteristic aromatic fragments with molecular weights of 3, 30, and 200 kDa had arisen in both cases. The increase of high-molecular-mass fragments (30 and > 200 kDa) is obviously due to a depolymerizing effect caused, *e.g.*, by *Xpo*GH78, which hydrolyzed ester bonds between the monolignol moieties of the lignin and the hemicellulose sugars. This would lead to a release of hydroxycinnamic acids with free hydroxyl groups, which can be further oxidized by laccase. Finally, this leads to further polymerization reactions and the formation of high-molecular-mass fragments. This effect was just observed by combining all enzyme preparations and points to a synergistic action by all of the biocatalysts used.

Moreover, the synergetic action of tested enzymes was proved by the release of *C*-5 and *C*-6 carbohydrates (glucose, xylose, galactose) from rape straw meal, which was enhanced by the use of either combination of all hydrolytic enzymes (Cell/Xyl and *Xpo*GH78) or additionally with *Xpo*Lac (although *Xpo*Lac alone did not affect the liberation of sugar from rape straw meal). Vancov & McIntosh [20] demonstrated the increase in sugar release from ~ 135 to 190 mg/g by doubling the reaction temperature from 60 to 121°C. Nevertheless, a higher reaction temperature (45°C) and chemical pretreatment were used in this study in contrast to the mild reaction conditions applied for this *Xpo*GH78-catalyzed conversion. The efficiency of enzymatic hydrolysis of lignocellulosic material depends on the enzyme amount or substrate used. Therefore, reaction parameters for the straw conversion by *Xpo*GH78 with and without accessory enzymes can be further optimized to improve the release of economically important metabolites.

Demethylation or pectin methylesterase activity of *Xpo*GH78 was evidenced by methanol release from the cell wall heteropolymer pectin. The enzymatic hydrolysis of ester bonds in the carboxymethyl group of the galacturonic acids in pectin may explain methanol release from the rape straw. The highest amount with approximately. 270 μg/g (≈ 27%) methanol was obtained using the enzyme cocktail containing *Xpo*GH78/*Xpo*Lac and Cell/Xyl. This finding may be of general biotechnological interest since the production of the so-called biomethanol from agricultural wastes could be used for the sustained synthesis of fuel supplements.

The role of the pectin methyl-esterase (PME) produced by phytopathogens during plant infection seems obvious: PME activity facilitates pectin degradation for the concomitantly depolymerizing enzymes and thus causes a synergistic breakdown of the cell wall barrier. The *Xpo*GH78 possesses the PME activity to hydrolyze methoxyl side chains of the pectic substrate as a cell wall component in rape straw. In the current study, the retardation of methanol accumulation by *Xpo*GH78 compared with the synergistic action of hydrolases and oxidase may reflect the inaccessibility of cell wall pectin methyl esters to sole *Xpo*GH78 action. Thus, addition of Cell/Xyl and *Xpo*Lac lead to a change in the cell wall structure and may influence the availability of pectinic substrate for PME activity of *Xpo*GH78 and in consequence, accumulation of methanol. Indeed, the esterase-catalytical conversions of methoxylated substrates such as phenylalkanoate methyl esters [21, 22] and complex lignocellulose substrates [23, 24] have been conducted, while the direct release of methanol from lignocellulosic materials by demethylating microbial hydrolases like XpoGH78 is still rare.

## Supplemental Materials

Supplementary data for this paper are available on-line only at http://jmb.or.kr.

## Figures and Tables

**Table 1:** Release of sugars from rape straw by enzymatic treatment.

Reaction system	Designation	Release of carbohydrates (mg/g)				

		Arabinose	Galactose	Glucose	Mannose	Xylose
Hydrolase	*Xpo*GH78	1.8	0.2	1.4	0.9	1.4
AB Enzymes	Cell/Xyl	1.3	0.5	4.7	2.3	3.4
Hydrolase &	*Xpo*GH78	2.5	1.5	5.5	3.0	3.5
AB Enzymes	Cell/Xyl					
Hydrolase &	*Xpo*GH78	2.3	3.0	5.9	2.6	3.5
AB Enzymes,	Cell/Xyl					
Laccase	*Xpo*Lac					
