# Supplementary material for: Bioconversion of Lignocellulosic Materials with the Contribution of a Multifunctional GH78 Glycoside Hydrolase from Xylaria polymorpha to Release Aromatic Fragments and Carbohydrates
Source: J Microbiol Biotechnol. 2021 Aug 20;31(10):1438–45. doi: 10.4014/jmb.2106.06053 (PMC9705965; doi:10.4014/jmb.2106.06053)

J. Microbiol. Biotechnol.
https://doi.org/10.4014/jmb.2106.06053

Table S1. Purification of FAE from the solid-state culture of *X. polymorpha*.

Purification steps	Total activity (U)	Total protein (mg)	Specific activity (U mg^{-1})	Yield (%)	Purification (fold)
Crude extract	57.4	910.8	0.06	100	1.0
Ultrafiltration	54.5	620.5	0.09	94.9	1.5
DEAE Sepharose	43.9	68.2	0.64	76.5	10.7
Superdex Sepharose	20.6	11.5	1.79	35.9	29.8
Mono Q	4.9	1.9	8.80	8.5	146.7

Figure S1. SDS-PAGE (left) and native IEF (right) of GH78 glycoside hydrolase of *X. polymorpha* (lanes 2 & 3) after SEC-HPLC; Protein maker (lanes 1 & 4).

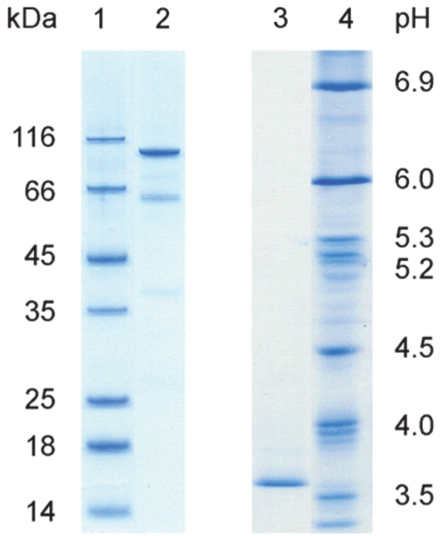

Supplement: Supplementary file 1 [file jmb-31-10-1438-supple.pdf]
